# Cetoleic acid and other long-chain unsaturated fatty acids as neuroprotective nutraceuticals

**DOI:** 10.1186/s12944-026-02876-8

**Published:** 2026-02-16

**Authors:** Patrik Anthony Tang, Maria José Ruiz-Pastor, Aurélia E. Lewis, Kari Espolin Fladmark, Asgeir Kobro-Flatmoen, Øyvind Halskau, Pradeep Lal

**Affiliations:** 1 Climate and Environment Division, NORCE Norwegian Research Centre, Bergen, Norway; 2 Physiology, Genetics and Microbiology, University of Alicante, Alicante, Spain; 3 Department of Biological Sciences, University of Bergen, Bergen, Norway; 4 Norwegian Health Association Centre for Dementia Research, Kavli Institute for Systems Neuroscience, Norwegian University of Science and technology, Trondheim, Norway

**Keywords:** Cetoleic acid, Erucic acid, Neurodegenerative disease, Alzheimer's disease, Lipid metabolism, Zebrafish

## Abstract

Long-chain monounsaturated fatty acids such as erucic acid, cetoleic acid and gondoic acid, are 20-22-carbon fatty acids with a double bond in their ω-9, ω-11 and ω-9 positions, respectively. Recent experimental research suggests that these lipids may provide benefits related to cardiovascular, but also brain health. Research on cetoleic acid using cell lines suggests that this fatty acid may positively affect neurological health. Also, in limited doses, erucic acid and gondoic acid have been reported to have a neuroprotective effect through action on peroxisome proliferator-activated receptors, and monounsaturated fatty acids generally are able to influence these receptors. Herein, we review the current state of knowledge of monounsaturated fatty acids effect on health, with an emphasis on erucic acid and cetoleic acid and their possible neuroprotective effects. Research has not progressed far regarding the direct neuroprotective effects of cetoleic acid, and mechanisms underlying such effects. However, both erucic and cetoleic acid influence the availabilities of docosahexaenoic and eicosapentaenoic acids, that do confer several health benefits, including neuroprotective effects. We highlight knowledge gaps related to metabolism, putative neuroprotective mechanisms, and briefly review animal model systems suitable for investigating these gaps.

## Introduction

Lipids are a diverse group of compounds that comprise fatty acids, waxes, sphingolipids, glycerophospholipids, di- and triacylglycerols, and sterols among others [1]. Their uniting characteristics are relatively low molecular weights combined with solubility in organic solvents but not water [1, 2]. Lipids have important energetic value and thus act as major energy sources, but also have critical structural and functional roles such as forming the lipid bilayer structures of cell membranes, or as physiological regulators by acting as cofactors for protein functions and cell signaling pathways [3–5]. Overall, lipids are of high nutritional value, and imbalances in their intake can lead to numerous detrimental impacts on health. For instance, it is well-recognized that the dietary provision of lipids is an important factor in the development and prevalence of modern day diseases, including the cardiovascular system (e.g., coronary disease) and metabolic syndromes (e.g., type 2 diabetes) [3, 6].

Lipids have also been implicated in neurodegenerative diseases such as Parkinson’s Disease, Huntington’s Disease, and Alzheimer’s Disease (AD) [7–10], the latter disease having received the most attention, and the main focus of this review. AD is a distinctly age-related disease whose prevalence doubles every five years beyond the age of 65, affecting 15–20% of people aged over 80. Aside from age, genetics contributes greatly to the development of AD, accounting for an estimated 60% of the disease risk [11]. The remaining 40% is thought to represent modifiable life-style risk factors, involving for example traumatic head injuries, smoking, obesity, diabetes, hypertension, depression, and diet [12–14]. Imbalances in the dietary provision of essential lipids and altered lipid processing have been proposed to be part of the risks leading to AD and, as such, specific lipids might be important for preventing or slowing disease onset. Supporting this, lipid replacement approaches have been reported to improve cognition in AD animal models as well AD patients [15–17].

It has long been known that sufficient intake of omega-3 fatty acids with long, polyunsaturated fatty acids (PUFAs) such as docosahexaenoic acid (DHA) and eicosapentaenoic acid (EPA), as well as limiting the intake of cholesterol have positive health effects in both animal models and humans [3, 18]. Furthermore, keeping the ratio of saturated (SFAs) to monounsaturated (MUFAs) and PUFAs to 1:1.5:1 is recommended by the World Health Organization [19]. The risks associated by non-optimal lipid intakes are related to the development of obesity, diabetes, cardiovascular complications, and neuroinflammation, health issues that overlap considerably with the known risk factors for AD [13, 20].

**Fig. 1:**
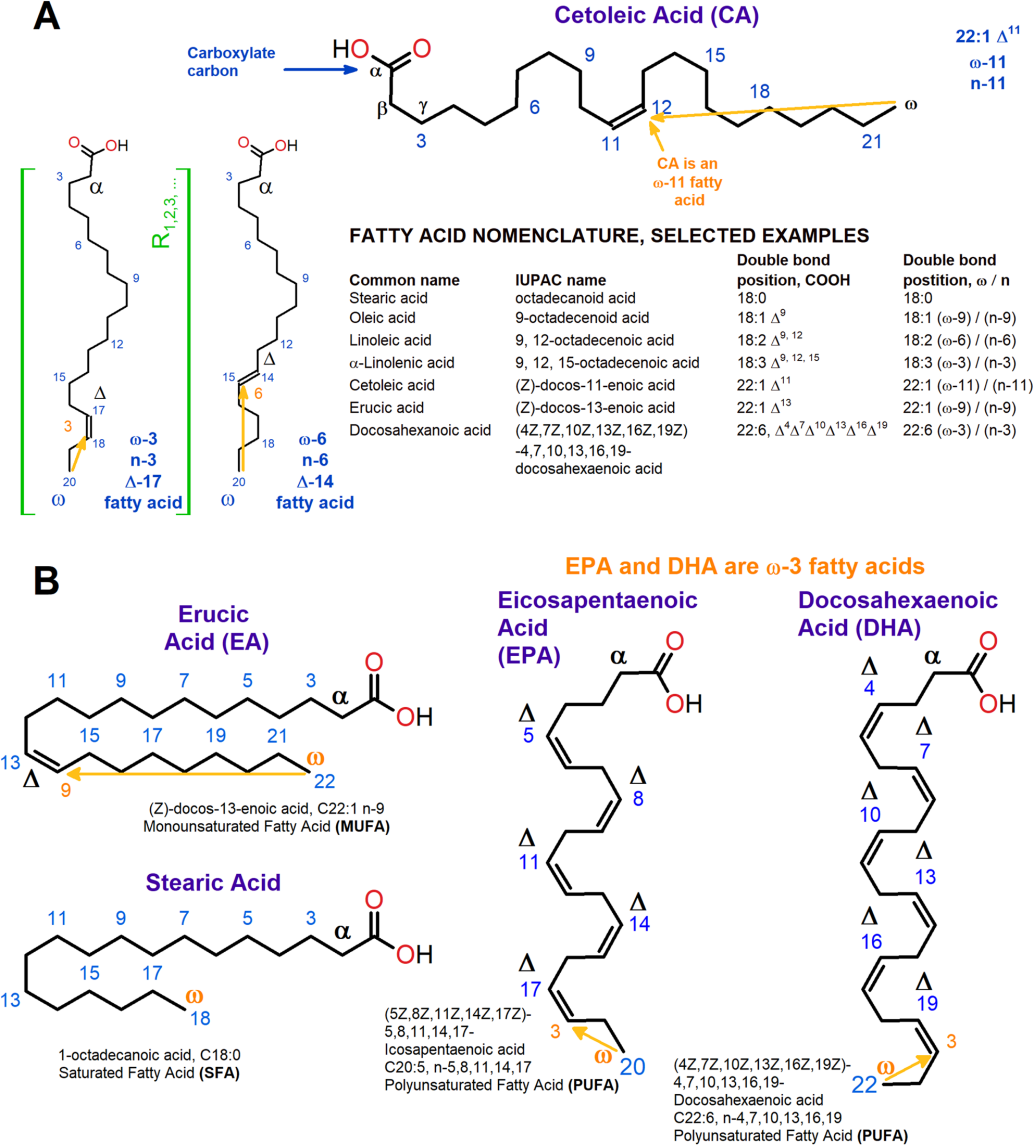
Long chain mono- and poly-unsaturated fatty acids -, and their lipid nomenclature. **A** Structure of cetoleic acid and selected lipid nomenclatures relevant for this review. Carbon atom numbering in blue is counted from the carboxyl carbon and is used to denoting the Δ-positions of the carbon double bondsrelative to this position. Another numbering scheme, indicated with yellow numbers, counts from the terminal methyl carbon, or ω-position/n-position, to the first carbon in the double bond. The α, β and γ positions near the carboxyl carbon are also indicated. **B** Representative structures of saturated fatty acids, monounsaturated fatty acids and polyunsaturated fatty acids. Erucic acid, stearic acid and two important polyunsaturated fatty acids, eicosapentaenoic acid and docosahexaenoic acid are shown along with their systematic names. Figure prepared in ChemSketch 12.01 [26]

The effects of DHA and EPA on cognitive functions show that these lipids improve spatial memory and lowered depressive states [21, 22]; however, other fatty acids exist with potentially similar positive action that to date have received less attention and scrutiny. Cetoleic acid (CA), IUPAC name, (Z)-docos-11-enoic acid, C22:1 n-11, is a 22-carbon fatty acid that has a single cis double bond at position 11, and therefore belongs to long chain MUFA (LC-MUFA) class. The chemical structures of CA and a summary of lipid nomenclature can be viewed in Fig. 1A, while Fig. 1B shows erucic acid (EA), stearic acid and their chemical structures in relation to DHA and EPA. Marine oils of especially pelagic origin (e.g. zooplankton, herring) are rich sources of LC-MUFAs, especially CA. However, due to inherent metabolic constraints in humans to produce CA *de novo*, its dietary partitioning is likely key for its bioavailability. Similar to EPA and DHA, CA is associated with reducing several risk factors related to cardiovascular and metabolic health, and importantly involved in improving healthy ageing in humans [23–25]. Due to this importance for AD risk, this review outlines the possible direct and indirect actions CA and similar FAs might have on AD and its prevention, alongside potential molecular targets with special reference to the central nervous system (CNS).

## Cetoleic acid source and metabolism

**Fig. 2:**
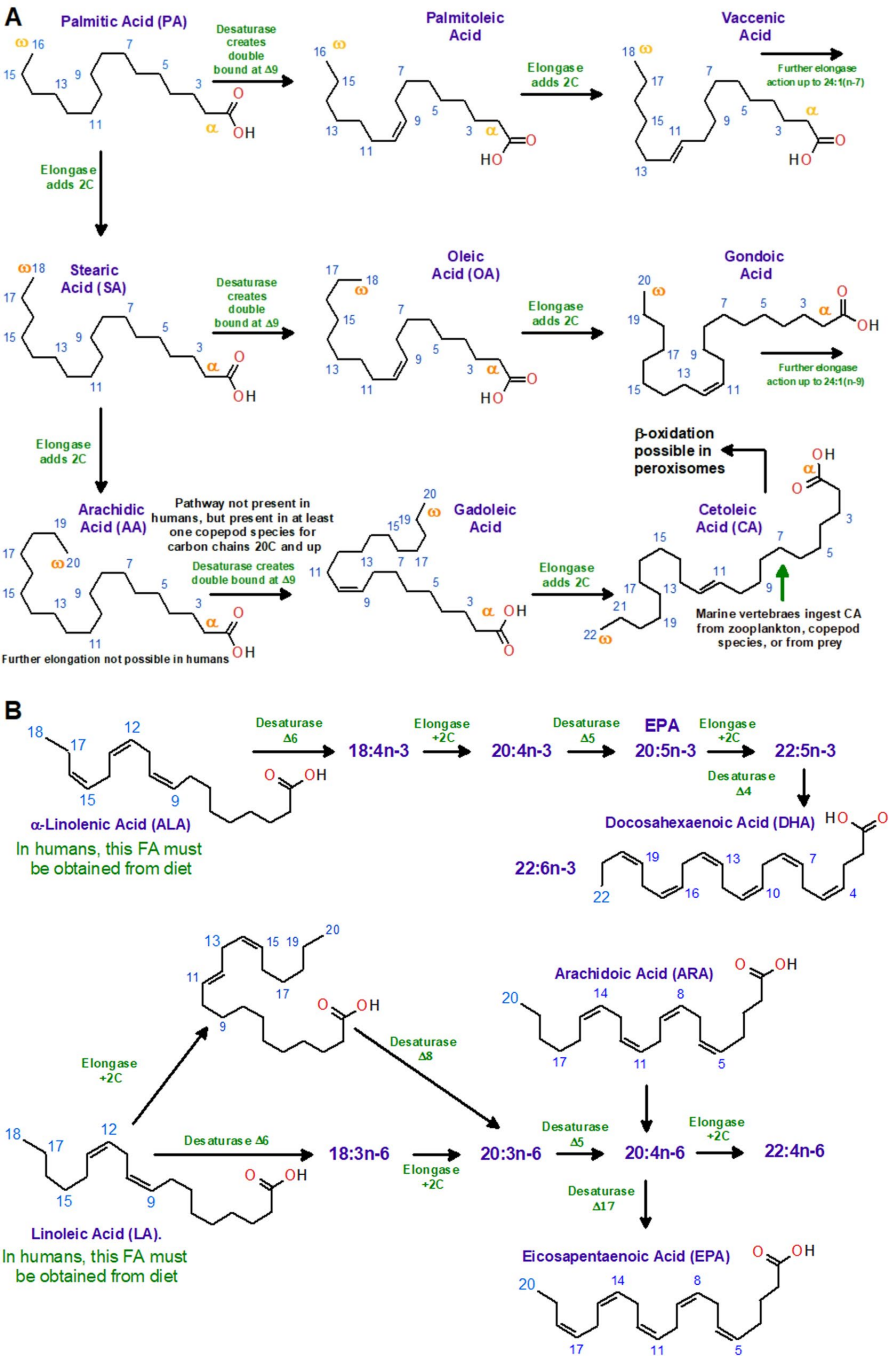
Selected LC-MUFA, LC-PUFA metabolic pathways. (**A**) LC-MUFAs and CA are generated by elongation of the saturated fatty acids, e.g. palmitic acid (16:0), to stearic acid (18:0), and arachidic acid (20:0). In the case of CA, desaturation on C9 by acyl-CoA Δ9 desaturase to gadoleic acid (20:1n-11) and an elongation to CA, 22:1n-11, results in this FA. In mammals, Δ9 desaturases are reported to use substrates with a maximum of 18 C and are therefore unable to synthesize CA. In marine vertebrates, CA is likely to originate from ingested zooplankton and copepods rich in wax esters with abundant of CA moieties [28, 30–33]. In mammals, arachidic acid cannot be elongated or desaturated further; cetoleic acid is obtained from diet only. Lipid groups. (**B**) Synthetic pathways leading to DHA and EPA [34]. In vertebrates, desaturation is only possible up until C9 due to their lack of desaturase with Δ12 and Δ15 activity [35, 36]. In humans, the precursors ALA and LA therefore be obtained from the diet (plants, eggs and meat being important sources) [37]. The dietary ALA and LA are then elongated and desaturated to n-3 and n-6 PUFAs, such as ARA, DHA and EPA. Blue numbers indicate carbon atom positions, counted from the carboxyl carbon. Figure prepared in ChemSketch 12.01 [26]

To some extent, CA is distanced from terrestrial vertebrate metabolism, and it is unclear how well it is incorporated into human fat metabolism. We have briefly summarized current knowledge on CA synthesis in Fig. 2A, as well as the important PUFAs DHA and EPA in Fig. 2B. Starting at towards the bottom of the food chain, CA forms large wax esters in zooplankton and copepods [27, 28]. Wax components where CA is a fatty acid substitution are then consumed and rise in the food chain, where the wax is separated into FAs, metabolized or incorporated into other lipids. Although not comprehensively researched, it may be speculated that CA influences other lipid’s metabolism by accumulating and interfering in conversion pathways. In mink and seals, dietary CA does not easily get incorporated in subcutaneous fat tissue, which suggests that it accumulates elsewhere due to lack of digestive and transportation pathways [29].

### Current basis for dietary recommendations

Most guidelines to reduce the risk of AD include having a varied diet rich in fish, cereals, fruits and vegetables, dubbed the MIND diet [38, 39], in which the presence of unsaturated fatty acids is thought to play an important role [40]. The effect of dieting upon the risk of dementia is nevertheless somewhat unclear. Whereas several studies have reported that adherence to the MIND diet is associated with a reduced incidence of AD and dementia [38, 39, 41–46], other studies have not found any effects on dementia associated with such a diet. However, a recent large meta-study including about 65.000 subjects found that following the MIND diet was indeed linked to a reduced incidence for dementia, including AD [47, 48]. The weight of the evidence therefore supports the notion that diet impacts the risk of AD and dementia, in line with the current recommendations from the World Health Organization [49].

Based on these recommendations, dietary supplements are one of the main options for the clinical management of patients with neurodegenerative diseases. Nutraceuticals containing oils enriched with omega-3 have gained popularity, but specific recommendations that directly involve LC-MUFAs such as CA are not in place. The specific source used to produce nutraceutical oils is not usually described. Fish oil is the most common source, but not all fish contain the same proportion of DHA, EPA or LC-MUFAs. While anchovies and sardines contain a high proportion of DHA and EPA, capelin, herring, and mackerel oil show a lesser quantity of n-3 LC-PUFAs and a higher proportion of LC-MUFAs. Among them, in herring oil, CA stands out with concentrations as high as 22% of total fatty acids [25], although this also varies with e.g. season. Some vegetable oils also contain high amounts of LC-MUFAs such as EA and gondoic acid, most notably oil from *Brassica nigra* (black mustard) seeds [50]. The EA content of the widely used rapeseed oil has largely been removed through breeding efforts since EA is perceived as health hazard when consumed in this product [51, 52]. However, these toxic effects, originally found in rats, are not evident in other relevant studies or in populations that consume large amounts of EA [27, 50, 52].

Unfortunately, there is a scarcity of research directly comparing fish oils with identical EPA and DHA levels but varying MUFA content. This makes it challenging to pinpoint the unique effects of MUFA *in vivo* and constitutes a gap in knowledge for those developing dietary recommendations, as well as a gap in information for producers and consumers of oil-based dietary supplements. It would be necessary to fill these gaps to take advantage of any benefits that such nutraceuticals could provide.

## Long-chain MUFAs, PUFAs and ω-3 fatty acids

Marine ingredients are rich sources of n-3 LC-PUFAs, such as EPA and DHA that are thought to provide health benefits related to the cardiovascular system, inflammation, pregnancy and cognition [22, 53–55]. However, the mechanisms by which these health benefits are exerted and related are still subject to discussion. For instance, traditional marine oil concentrates containing very high levels of EPA and DHA are reported to beneficially alter cholesterol metabolism in humans, while oils derived from e.g. pelagic fish sources attain the same physiological benefits despite containing considerably less EPA and DHA [56, 57]. This discrepancy is hypothesized to being due to differences in other important FAs that can vary between oil types, such as LC-MUFAs that are enriched in many pelagic fish species [24].

The LC-MUFAs cetoleic acid, gadoleic acid, gondoic acid and erucic acid can have direct bioactive effects on metabolism and overlapping functions with ω-3 PUFA pathways, providing health effects in humans [57]. For instance, these LC-MUFAs are noted to improve lipid and sterol metabolism in humans and other mammals [58], such as lowering LDL and total cholesterol (TC) via enhanced bile conversion as reported in hypercholesterolaemic rodents or during fat-rich diets [59, 60]. Others report improved retention of ω-3 PUFAs such as EPA and DHA when supplemented with CA [57]. Moreover, since LC-MUFAs such as CA cannot be synthesized by mammals, diet is essential in acquiring these bioactive FAs. However, dietary CA can still be metabolized in peroxisomes through β-oxidation to gadoleic acid, although generally this FA has poor digestibility [29]. Provided that the minimal dietary requirements of EPA and DHA are met, it is likely that CA-rich oil and its associated LC-MUFA composition could play important roles in modulating EPA and DHA related health benefits. This can be achieved either by indirectly altering their metabolism or by having direct complementary health benefits. Still, such effects remain poorly understood for most fatty acids and undocumented in the case of CA. In one of the few direct studies specifically exploring CA health effects in humans, improved endothelial function and increases the efficiency of ALA conversion to DHA and EPA when available in abundance [23].

### Effect of LC-MUFAs on cardiovascular and metabolic disorders, and related blood risk factors

Evidence is accumulating that dietary LC-MUFAs may have preventative properties in many cardiovascular and metabolic disorders (e.g., atherosclerosis and type 2 diabetes mellitus (DM)). The first epidemiologic evidence of this was observed in Greenland Eskimos, which revealed a link between LC-MUFA (and also ω-3 PUFA) consumption and cardiovascular health and protection against cardiovascular diseases [61]. In agreement, the Physicians’ Health Study showed an inverse relationship between red blood cell LC-MUFA content and incidence of cardiovascular diseases, supporting potential atheroprotective effects [62]. Similarly, a series of experiments by Yang and colleagues showed in mice that dietary saury oil and pollock oil rich in LC-MUFAs, provided hypolipidemic and anti-inflammatory effects by upregulating PPAR and curbing inflammation, aspects critical to reduce risks related to atherosclerosis and coronary heart disease [23, 61]. This was confirmed in approaches using LC-MUFA concentrates that improved endothelial function and reduced the development of atherosclerosis lesions alongside several plasma pro-inflammatory cytokines in mice models [23, 30], while higher doses further attenuated pro-atherogenic plasma lipoproteins and atherosclerosis in ApoE-KO mice [30].

Evidence supports dietary FAs also affecting insulin sensitivity and glucose/lipid metabolism [63]. In contrast to SFA, MUFAs are increasingly accepted as being protective against obesity and DM [64]. In line, LC-MUFA rich oils reduce plasma glucose and lipid levels, leading to less hyperlipidemia and steatosis in obese mouse models [23, 30, 61]. Likewise, LC-MUFA oils reduced adipocyte size, white fat mass and plasma lipid, insulin and leptin levels in type 2 diabetic KK-Ay mice. These oils show to improve lipid metabolism indicators commonly associated with ameliorating dysfunctions related to DM [27, 59]. Key genes related to lipid metabolism in adipocyte tissue, including PPARγ, lipoprotein lipase, FATP, fatty acid translocase/CD36, palmitoyltransferase-1 A, and citrate synthase were affected [27].

In particular, LC-MUFAs are reported to alter the blood profiles of low-density lipoprotein (LDL), high-density lipoprotein (HDL) and triacylglycerol (TAG) levels that are commonly associated risk factors for the development of disease [24]. For simplicity we will concentrate on two primary LC-MUFAs, CA and EA. CA is reported to reduce blood LDL levels by improving LDL receptor activity, its blood clearance, and lowering overall hepatic LDL production [59]. Further, evidence suggests that CA has mildly positive effects on HDL levels, similar to other MUFAs like oleic acid (OA, 18:1n-9) in enhancing blood HDL content [65]. Conversely, CA intake is also associated to reducing blood TAG levels by promoting energy and oxidative pathways that inhibit hepatic TAG synthesis and stimulate its breakdown [57, 59, 66]. On the other hand, the effects of EA blood profiles are conflicting, especially in regard to the reported toxicity of EA rich oils. It is indicated that since EA metabolism is slow [67], consumption at high amounts risk disrupting TAG metabolism and elevating dyslipidemia, resulting in unfavourable sterol metabolism, hepatic steatosis and elevated blood LDL levels [68]. Therefore, high EA intake may be less favourable compared to CA or OA. In fact, excessive intake of EA is also linked to myocardial lipidosis, raising cardiovascular concerns [68]. In all, with these toxicity effects of EA reported in many animal models, safety recommendations for human consumption still requires clarification. Conversely, as outlined in Table 1, CA seems more beneficial in stimulating healthy lipid metabolism compared to EA. Still, due to our incomplete understanding over the direct evidence regarding the precise mechanisms and extent of LC-MUFAs’ functions, further study is needed to ascertain the risk factors relative development of neural and metabolic disorders.

**Table 1:** Summary of effects of cetoleic acid and erucic acid on blood LDL, HDL and TAG levels

Fatty Acid	LDL	HDL	TAGs
Cetoleic Acid (22:1n-11)	No effect or ↓ [58]	No effect or ↑ [66]	**↓ **[58, 60, 67]
Erucic Acid (22:1n-9)	**↑**	No effect	↓ Risk of liver steatosis

### Metabolic interplay between LC-MUFAs and n-3 PUFA metabolism

**Fig. 3:**
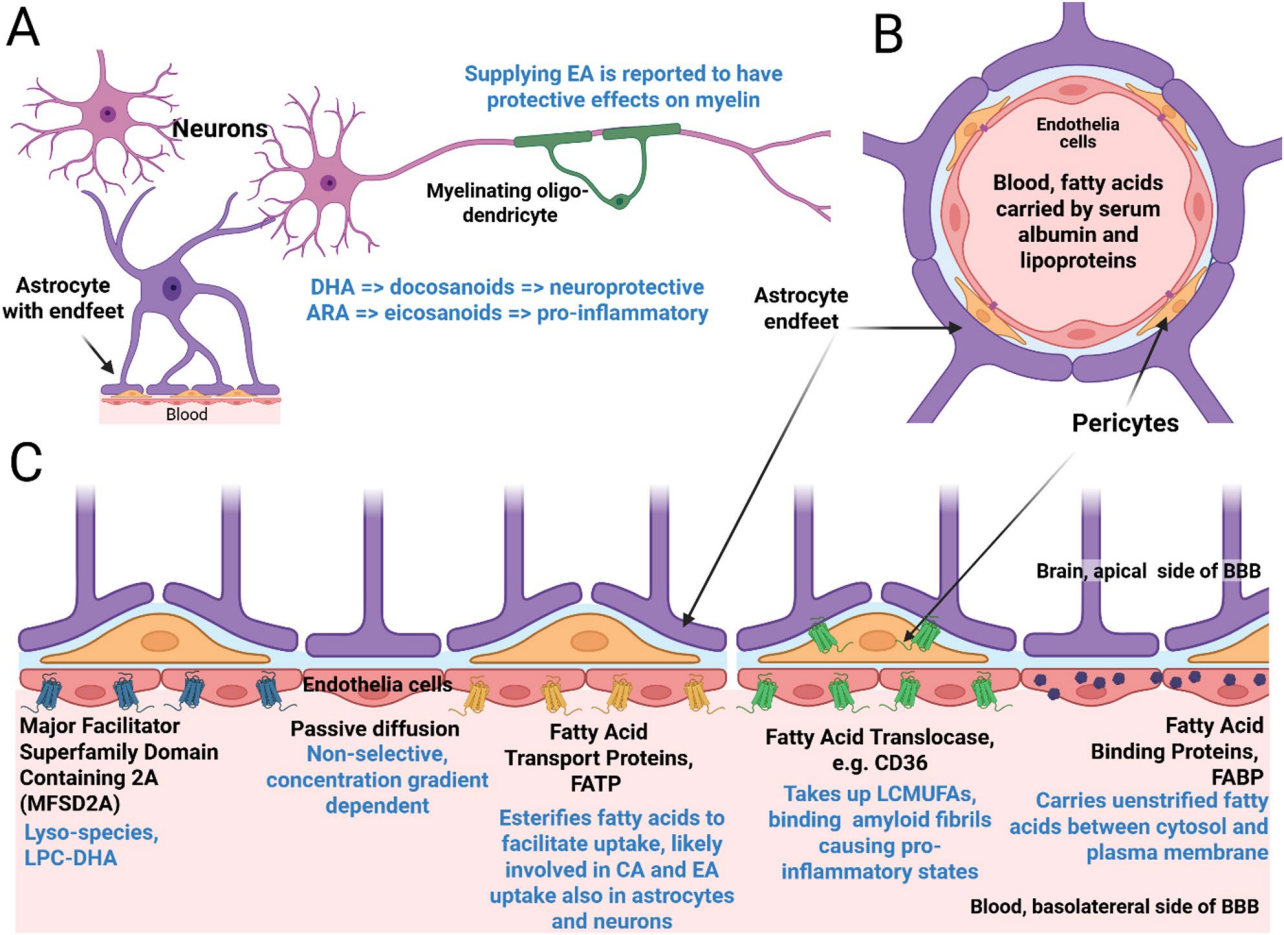
The CNS, Blood Brain Barrier and associated lipid transport factors. (**A**) Neurons, astrocytes and oligodendrocytes, and their relation to the Blood Brain Barrier. Supplying EA is reported to have positive effects on myelination [82]. CA and EA levels may have effects on DHA and ARA-derived docosanoids and eicosanoid levels, which are viewed as neuroprotective and pro-inflammatory, respectively [83, 84]. (**B**) Cross section of Blood Brain Barrier (**C**) Lateral view of the human blood brain barrier. Cell types and important proteins involved in FA transport across the barrier or for uptake (black font), as well as lipid subtypes (blue font) associated with each mechanism indicated. https://www.biorender.com/ MFSD2A, FATPs, and Fatty Acid Translocases/CD36 are all transmembrane proteins that act as flippases to bring fatty acids to the other side of the plasma membrane they are embedded in [85, 86]. Some FATPs may also modify fatty acids to CoA-fatty acid conjugates [85], priming them for β-oxidation or incorporation into other lipid molecules. The FABPs are not membrane spanning, but globular proteins that exist in both cytosolic and plasma membrane associated forms [86, 87]. Although shown exclusively in Panel C, these proteins also help uptake of lipids after the endothelia cells of the BBB are crossed, and are expressed in neurons, pericytes and astrocytes to varying degrees. Figure created with https://www.biorender.com/

In mammals, the conversion efficiency of ALA to EPA and DHA (Fig. 3B) *in vivo* is poor, however, distinct factors can modulate this capacity, including diet, developmental stage, genotype, and growth [69]. Especially, dietary fatty acids can exert large consequences on ω-3 PUFA conversion efficiencies, effects well-described for the dietary intake of ALA, EPA and DHA. For example, excessive intake of DHA can inhibit ω-3 biosynthetic pathways [55] while dietary restrictions in DHA stimulate these pathways [70]. Moreover, evidence is accumulating that other dietary FAs, including LC-MUFAs, might play important roles in stimulating ω-3 PUFA biosynthesis and metabolism [57]. While LC-PUFAs are generally more effective activators of the PPAR proteins [71, 72], there are indications that LC-MUFAs, such as CA, might also alter the expression of key proteins involved in lipid metabolism, including PPARs and acyl-CoA oxidase [73, 74]. In agreement, LC-MUFAs have been noted to affect nuclear receptors, such as PPAR-α, PPAR-δ and PPAR-γ [30, 59, 75] that may transcriptionally alter oxidation pathways, LC-PUFA biosynthesis, and their balance [76]. However, whether these act as transcriptional regulators of the expression and activity of elongases and desaturases critical in PUFA biosynthesis pathways is still unknown, warranting examination. Nevertheless, because CA is reported to stimulate the β-peroxidation for energy [77], with this metabolic pathway being closely linked to the biosynthesis of ω-3 PUFAs [78], it is not inconceivable that diets rich in CA may benefit the production of DHA [79]. In line, such effects of CA have been observed in human and salmon hepatocytes *in vitro* and salmon *in vivo* [80]. If true, this would be favorable for ω-3 PUFA metabolism with noted benefits on human health, particularly in relation to physiologically important FA ratios (e.g., ω-3/ω-6; EPA/ARA). For instance, several studies substantiate the benefits of EPA and DHA derived metabolites termed “lipid mediators” in curbing the negative effects of inflammation by the production of anti-inflammatory lipid mediators and specialized pro-resolving mediators [30, 54]. Naturally, such aspects are at the core of preventing the development of neurodegenerative diseases such as AD, as well as many cardiovascular conditions [81]. Nevertheless, this effect of CA has yet to be demonstrated in other organisms than rodents, warranting further study using complementary animal models.

## The blood brain barrier, cetoleic acid and erucic acid

Fatty acids required from dietary sources are transported in the blood stream either bound to serum albumin or lipoproteins [76, 88]. They must, to reach the CNS, pass through the blood-brain barrier (BBB, Fig. 3), and do so either by passive diffusion [89, 90], or by specific carrier mediated transport [89, 91]. Passive diffusion largely depends on the concentration gradient. Proteins of interest in active transporting across the BBB are the Fatty Acid Transport Proteins (FATP). These are very long chain acyl-CoA synthases that helps fatty acid uptake via esterification-coupled influx of already internalized fatty acids either by passive diffusion or by specific carrier protein [92]. In addition to FATPs, Fatty acid translocase (CD36) and fatty acid binding proteins (FABPs) are some of the key transport genes involved in transport of fatty acids across BBB and are highly expressed in the endothelial cells in the BBB [89, 92]. FABPs [87], CD36 [85], FATPs [86], as well as blood lipid transporter Apolipoprotein E [44], are all implicated in AD.

Long-chain fatty acids likely require an active uptake mechanism. Specific proteins involved in the transport of specific lipids are not always known for every fatty acid or lipid in question, and this is the case for EA and CA. It is likely, however, that the FABPs play a role as they are known to work on LC-MUFAs. There are currently no studies on direct uptake of CA through the BBB. However, an isotope-labelling study on the very similar fatty acid EA has shown that plasma derived EA can cross BBB and be incorporated into specific lipid pools in the brain where it undergoes β-oxidation [67]. After entering the CNS fatty acids are further metabolized in neuro-supportive astrocytes and oligodendrocytes [93].

Modification of fatty acids to specific forms can allow transport across BBB. Major Facilitator Superfamily Domain Containing 2 A (MFSD2A), is a BBB transporter expressed exclusively in endothelium of the blood-brain barrier of micro-vessels; MFSD2A has been linked to dementia and a deficiency of MFSD2A leads to leaky BBB and to cognitive deficit and developmental defects in mice [88], zebrafish [94], and humans [95]. MFSD2A is known to transport common plasma lysophosphatidylcholines (LPCs) carrying long-chain fatty acids such LPC oleate and LPC palmitate, but not LPCs with less than a 14-carbon acyl chain; it facilitates transport of DHA in the form of lysophosphatidylcholine (LPC-DHA) [88]. There is also evidence that lyso-species are transported across the BBB as exosomes [96]. However, to what extent EA or CA-containing phospholipids are converted into lyso-species and eligible for crossing the BBB through this mechanism is currently not known.

### Direct effect on structure and function of neurons

Neuronal membranes are enriched in highly specialized lipid species that determine the biophysical properties of the lipid bilayer. However, neuronal lipids also act as reservoirs of secondary messengers that influence a myriad of routes or even determine neuronal survival [10, 37]. Recent research has shed some light on the nature and molecular mechanisms of these metabolites, expanding our understanding of lipid pathways in the nervous system and suggesting new possibilities for neuroprotective therapies [17, 83, 97]. For example, palmitoleic acid (C16:1 n-7) has been described as an anti-inflammatory lipoine that also prevents endoplasmic reticulum stress in adipose tissue [98], while hypogeic acid (C16:1n-9) has been implicated in regulating metabolism in adipose tissue [98]. Supplying EA, known for its antioxidant and anti-inflammatory effects, has been demonstrated to improve cognitive function and protect myelin in Parkinson’s disease [82]. While questions remain, these findings are of clear importance for AD.

Current knowledge concerning CA in neurons is limited. Østbye and colleagues showed that CA promotes the conversion of ALA to DHA and EPA in HepG2, a human hepatocyte-derived cell line, and to EPA in primary salmon hepatocyte cells [80]. ω-3 fatty acids and, particularly, DHA, are key components for the development and homeostasis of the neurons. Scientific literature agrees that higher circulatory levels of ω-3 fatty acids in patients are related to a better outcome of disease, meanwhile, an increase in the ratio ω-6/ω-3 promotes an inflammatory state [99, 100]. DHA and ARA, which are ω-3 and ω-6 PUFAs, respectively, are released from the lipid bilayer by the action of phospholipase A2. In the cytosol, the free fatty acids are metabolized by different lipoxygenases and cyclooxygenases, resulting in the docosanoids, in the case of the metabolites from DHA, and eicosanoids, from ARA [84]. Docosanoids are anti-inflammatory and anti-apoptotic, while most eicosanoids are pro-inflammatory [101]. In both animal models and patients with neurodegenerative diseases, lower levels of DHA were found in neural tissues and blood [102–104]. In this context, CA could represent an interesting candidate for neuroprotective therapies to increase the synthesis and retention of DHA, and herein, raise the production of docosanoids in animal models and patients.

An important family of fatty acids for the structure of neural membranes are the very long-chain fatty acids, which are formed through different steps of elongation of long-chain fatty acids. Very long-chain fatty acids are especially abundant in synapses and other highly specialized lipid bilayers found in certain neurons. For example, the structure and fluidity of the disc membranes of the outer segments of photoreceptor cells are supported by very long-chain fatty acids, as is the conformational changes of transmembrane proteins [105]. The nature, the pathways of synthesis, and the biological roles of different saturated and polyunsaturated very long-chain fatty acids in the brain and the retina have recently been described [106]. In contrast, little has been reported specifically regarding very long-chain MUFAs, such as CA. It is therefore currently not certain whether CA, with a hydrocarbon chain of 22 carbons, could serve as a substrate for elongases and be incorporated into very-long chain MUFAs, nor are transport and uptake mechanism clear. More research combining lipidomics with transcriptomics and proteomics, as well as targeted functional assays, is needed to elucidate the roles of CA and other MUFAs in neurons.

## The effects of ω-3 fatty acids on amyloid-β

The generation of amyloid-β (Aβ) by protease action on the amyloid precursor protein (APP) is a much-investigated molecular mechanism related to AD. APP is a membrane protein with an exposed feature that is targeted by proteases called α-, β- and γ-secretases. Normally, the exposed feature is cleaved off into fragments, first by α-, β-secretases, then γ-secretase [107]. The products are functional and not prone to amyloid formation. However, upregulated β-secretase, also referred to as BACE1, can overproduce amyloidogenic peptides, of which Aβ40 and Aβ42 are the most common components of the resulting amyloid plaque and associated with disease progression [107, 108]. Given that the production of Aβ takes place at the membrane, it is not unexpected that there is evidence for links between AD and lipid metabolism [109, 110]. There is also growing evidence that lipids can have a suppressive effect on the accumulation of misfolded Aβ aggregates. And, as noted in the section on the BBB, Aβ-aggregates may affect lipid transport factors and cause pro-inflammatory states [85].

To the best of our knowledge, no published studies have examined the possible impact of CA on Aβ, or Aβ impact on CA transport and uptake. Just a few studies have been conducted on the chemically similar EA and gondoic acid (Figs. 2B and 3A). In one study [50], AD was induced in rats using Aβ42 injection. AD rats fed oil from *Brassica nigra* seeds, showed better cognitive function relative to the control group. The *Brassica nigra-*oil contained approximately 25% and 17% percent of EA and gondoic acid, respectively, ND the third most abundant oil component being 8,11-Octadecadicnoic acid (~ 15%). The study discussion suggests that the molecular effects seen may be similar to that of oleic acid acting on Protein Kinase C and causing improved synapse health and long term potentiation [50, 111].

EA is also targeting PI3K, ERK, CREB and PPAR isoforms, particularly PPARẟ, and have been proposed to have antioxidant and anti-inflammatory effects relevant for neurodegenerative diseases [75]. Moreover, EA seems to have direct effect on Aβ-production. In a fairly recent study [112], investigators supplied EA to cell models for AD through complexation with bovine serum albumin. They found that EA was incorporated into cellular membranes, which became measurably thicker. They also showed that this thickness altered the output of Aβ-species from APP processing by γ-secretase [112], with increased amounts of especially the shorter Aβ38 and Aβ37 species which are linked to less amyloid aggregation and better clinical outcomes [113].

EA findings are similar to the much more extensive research conducted on DHA and EPA. Experiments on rodents that were chronically infused with Aβ40 into the lateral ventricles found that a diet high in DHA counteracted the detrimental effects of the treatment, as assessed by a simple memory task. This was linked to a reduction in reactive oxygen species (ROS) levels presumably caused by the Aβ40-infusion [114]. In line with this, more recent work using immortalized mouse microglia found that DHA suppressed the capacity of synthetic oligomeric Aβ species to induce ROS, possibly by upregulating the antioxidant related erythroid 2-related factor-heme oxygenase 1 (Nrf2-HO1) pathway [115]. By use of rodent models, DHA has also been linked to reduced Aβ42 levels through activation of the serine/threonine-protein kinase ULK1 autophagy-inducing pathway, most notably by increasing autophagosome-lysosome fusion [116]. Further experimental findings indicate that DHA supplementation could lower Aβ-levels also by reducing the levels of the presenilin 1 component of γ-secretase [117] or reducing the activity of both β- and γ-secretase in favor of increased α-secretase activity [118]. Additionally, in one cell culture-based study, both DHA and the related EPA apparently reduced Aβ-levels by affecting the Aβ-degrading enzyme insulin degrading enzyme [119]. However, in a more recent study using the triple-transgenic 3xTg mouse model, one of the most commonly used AD animal models, the authors reported that providing the animals with diets enriched in either DHA or EPA had no effect on Aβ-levels as measured in the parieto-temporal cortex [120]. Based on the evidence, it seems likely that dietary fatty acids may reduce levels of Aβ and/or ameliorate parts of the detrimental effects of Aβ through several mechanisms. LC-MUFAs are understudied in this regard compared to PUFAs.

## Animal models for exploring LC-MUFA effects

**Fig. 4:**
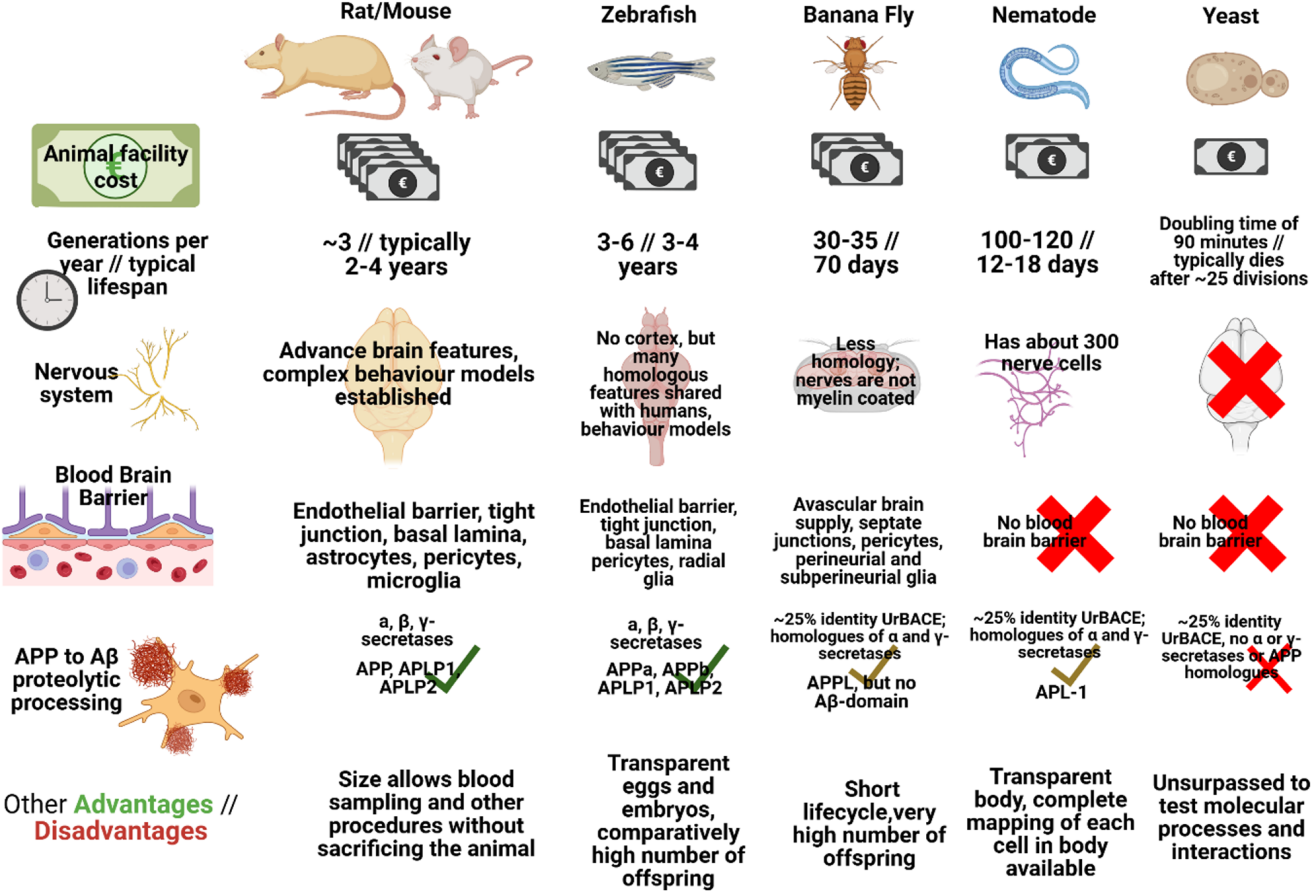
Visual summary of selected model systems and their strengths and limitations for investigating CA and LC-MUFA effects on Alzheimer’s Disease. Sources used to compile and curate the information presented in each row are as follows: Nervous system [121, 124–126], Blood Brain Barrier [125–127], Proteolytic processing [124, 128–130], Other Advantages/Disadvantages [121, 131, 132]. The rows concerning costs, lifetime and generations per year were based on accessible biological knowledge and verified in conversation with specialists. Figure created with https://www.biorender.com/

Investigating the potential neuroprotective nature of LC-MUFAs in general and CA in particular would ultimately involve time- and resource intensive studies with human subjects while adhering to strict ethical standards. As with other health-related research, investigations will therefore benefit from using animal models. Commonly used research animals for studying neurodegenerative diseases are the rodents *Rattus norvegicus* and *Mus musculus*, the nematode worm *C. elegans*, the fruit fly *Drosophila melanogaster*, and zebrafish *Danio rerio*. All have genetic models aimed at mimicking different metabolic and neurological diseased states, and are reviewed extensively by experts of their uses [21, 121–124]. In this short comment on their applicability for the topic of this review, we argue that the zebrafish may be a good choice among many excellent models. A visual summary of each model system’s characteristics in the context of this review is attempted in Fig. 4.

Being mammals credited with complex social behavior and advanced task-solving ability [133, 134], rodents have obvious advantages when studying brain function and dysfunction. Indeed, connectivity mapping of genes expressed in the two species indicate that e.g. the human cerebral cortex and mouse isocortex are similar, as well as their cerebellar hemispheres [127]. While a rather distant relative to humans, zebrafish are still considered a genetically tractable vertebrate suitable for neuroscience research. Behavioral assays for assessing sensory and cognitive performance are well established [135], and it in use for studying ageing [136]. Its brain, while simpler than a rodent’s, comprise features homologous to the human brain such as the corpus cerebellum, medulla oblongata, olfactory bulb, thalamus, hypothalamus, olfactory cortex amygdala, hippocampus and spinal cord, while lacking a cerebral cortex [137]. Also, given the role of the blood brain barrier in brain health and lipid transport [125, 138], the different or even lack of a blood-brain barrier in non-vertebrates may be a valid reason to pick the zebrafish, rather than invertebrate models (Fig. 4).

The ease of which it is possible to establish transgenic lines for model organisms is of obvious importance when choosing a model system. Establishing transgenic zebrafish lines is easier relative to rats and mice, because it provides a very large number of eggs after each mating. However, non-vertebrate models, *Drosophila melanogaster*, *C. elegans* and the non-animal model baker’s yeast, are easier still. These models all have impressive contributions to the study of diseases, including metabolic and neurological disorders [21, 124, 131]. Their sizes and biology make them even easier to work with at the cost of fewer relevant anatomical, physiological and genetic features, as well as shorter lifespan. The latter could be considered relevant, since AD develops with age, and so a short lifespan may not be sufficient to catch slow processes related to e.g. protein misfolding and gradual failure of the proteostatic network. Also here, the zebrafish may be considered attractive, as its lifespan is similar to rodents (Fig. 4).

### The zebrafish as a model system for Alzheimer’s Disease and lipid metabolism

The zebrafish underwent a genome-wide gene-duplication resulting in more than 26 thousand protein-coding genes and contains a high number of species-unique genes. However, while about 74% of human genes have one or more zebrafish orthologue [139], the relevant genes for AD and many other diseases are not all duplicated. The genetics of AD often focus on α-secretase (ADAM10), β-secretase (BACE1) and γ-Secretase, as well as APP. These are all present and conserved differently across organism [128, 129, 140] (Fig. 4). Depending on which aspects of AD are being investigated, this might disqualify some of the models. How well the disease-related amyloid pathway is mimicked in possible transgenic lines is outside the scope of this review, other than noting that rodents and zebrafish all have copies of APP variants, as well as all the secretases [107, 129, 140].

When studying LC-MUFAs and CAs neuroprotective effects it is also necessary to consider the animal model’s metabolism, especially how lipids are handled. Here, rodents have a disadvantage in that they do not always model human responses in response to high lipid diets [123, 132], and genome-wide mapping of human and rat genes metabolic genes find differences in bile acid synthesis and recycling, as well as sialic acid synthesis [141]. Moreover, rodents must spend energy to maintain their body-temperature [142], something the poikilothermic zebrafish does not need to do. At the same time, zebrafish is a well-established model for risk factor diseases including diabetes and obesity [143, 144], cardiovascular disease [145], hypertension, traumatic brain injury and neuroinflammation [121], and depression [135].

**Fig. 5:**
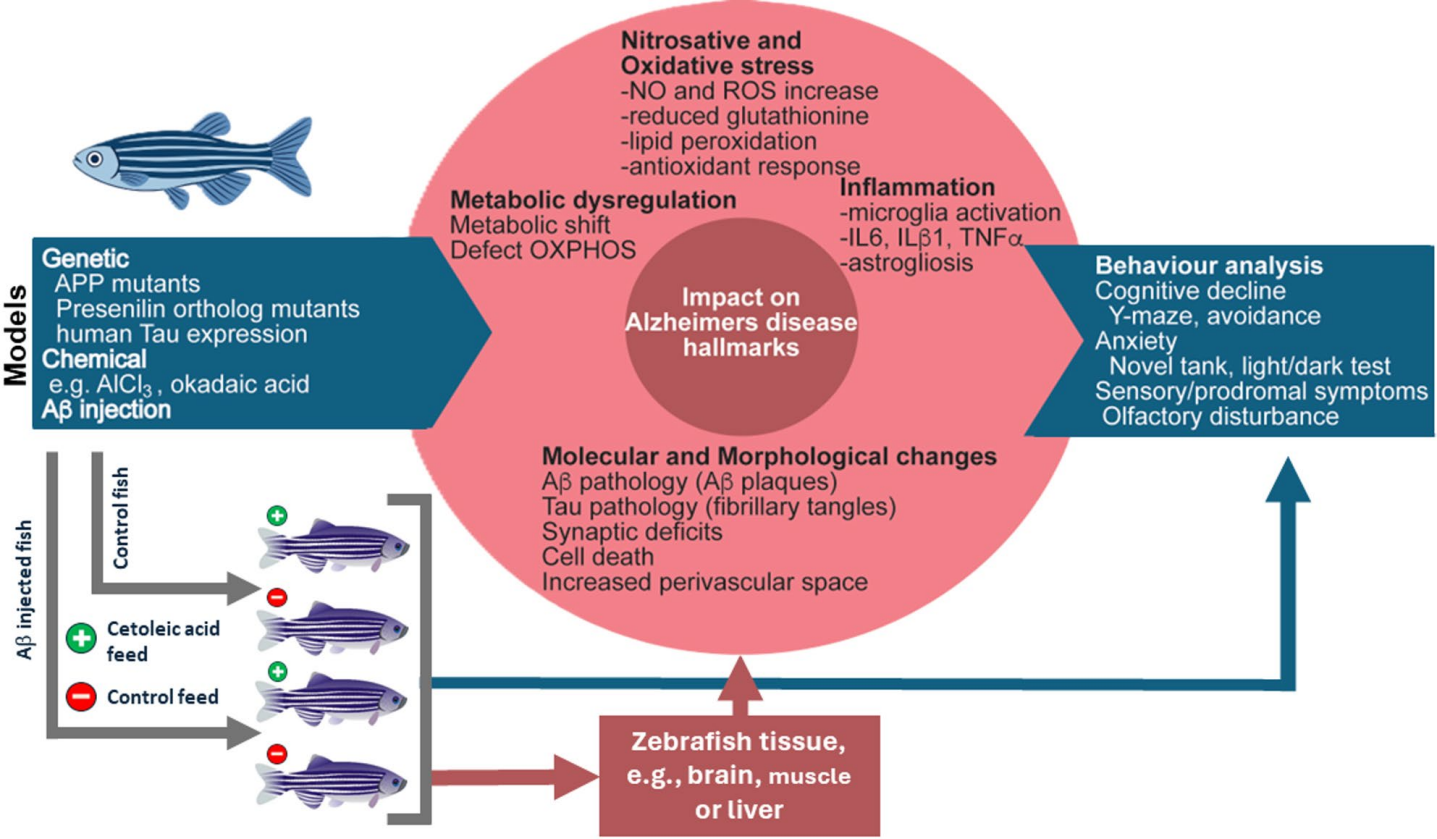
The zebrafish and its Alzheimer’s Disease-inducible models and how they could be applied in an experimental setup aimed at exploring the effect of cetoleic acid or other LC-MUFAs. AD can be induced by genetic modifications such as mutation of APP/presenilin genes, or inducing human Tau expression, by chemical stressors, or injection of Aβ. Selected metabolic, molecular, and cellular hallmarks of AD can be assessed using analytical techniques and assays developed for each marker. Alternatively -omics-type approaches can be adapted where this is possible and desirable, e.g., proteomics, metabolomics and lipidomic analyses for assessing metabolic shifts or changes in protein levels. Live fish can be assessed by a range of established behavioral assays, as indicated. At the bottom of the figure is a generic experimental setup where a zebrafish AD model and a control group is fed either a high cetoleic acid diet, or a control diet

The size and morphology of the chosen model will also impact which experiments can be undertaken. A great advantage of zebrafish compared to rodents, are that their eggs and early embryonic stages are transparent, and can therefore easily be imagined without sacrificing the animal [146]. On the other hand, the small and fragile zebrafish can make tasks such as blood collection difficult, and a definite advantage with using rodents is the possibility of monitoring e.g. blood across time without sacrificing the animal. Fig. 5 summarizes different approaches to modelling AD using zebrafish as a research animal, relevant molecular and behavioral outcomes that can be measured, as well as a proposed experimental setup for exploring the possible neuroprotective effects of CA.

The choice between model organisms inevitably represents trade-offs between advantages and disadvantages, and all have unique biologies. Yet, we view the zebrafish as an attractive choice as a model system for investigating the neuroprotective effects of CA, LC-MUFAs and more generally lipids. Findings from zebrafish research may complement existing knowledge derived from rodents and clarify issues that may be occluded by rodent-specific lipid metabolism. Moreover, while the zebrafish is somewhat simpler in terms of the nervous system, it still has a blood brain barrier and life-span similar to rodents, well-established behavioral models and preserved genetic features relevant to e.g. AD and other neurological diseases. At the same time, they are significantly easier to work with and cheaper to maintain than rodents.

## Future outlook

The health effects of LC-MUFAs such as CA and EA, while studied, are not understood well at a mechanistic level. As is evident from reading this work, knowledge specifically on CAs health effects and its underlying molecular responses are currently lacking. This may soon change. As of late 2025 there exists a number of registered clinical trials examining health effects of cetoleic acid and marine oils containing high concentration of cetoleic acid. Studies using fish oils concentrated with cetoleic acid and in some cases gondoic acid are exploring effects on skin function [147], atopic dermatitis [148], atherosclerosis [149], general health effects [150], and concentration of LDL cholesterol in adults [151]. Similarly, Calanus oil supplementation is being explored for its effect on maximal oxygen uptake [152], and the intra-abdominal fat, glucose tolerance and lipids in man [153].

CA’s proposed health effects may manifest either directly or through modulation of EPA and DHA. To differentiate between direct and indirect effects, it is necessary to run quantitative lipidomics on the amounts of DHA and EPA in test animals administered CA. EA, chemically similar to CA, has notable effects on PPARα, β, and γ , and CA should therefore be systematically investigated with respect to these targets. The result should be compared to PUFAs and MUFAs known to target the PPARs. Inflammation is part of the pathway to AD, and CAs anti-inflammatory effect should be investigated in animal models and humans by measuring specialized pro-resolving mediators such as annexin A1, interleukin 1 and 10, as well as glucocorticoids. Zebrafish models systems can accommodate investigation of all these markers (Fig. 5). The relevance of the blood brain barrier for brain health, neuroprotection and specifically AD is obvious; however, whether CA can be effectively transferred is not positively known, nor is the uptake of CA in e.g. astrocytes and neurons. In addition to FA transporting proteins, transport mechanisms may involve CA or EA being carried as part of lyso-phospholipids. It is also likely that amyloid aggregation may perturb fatty acid transport pathways, i.e., providing a feedback effect where healthy lipid uptake is compromised as the disease progresses. Although all commonly used model systems for biomedical research will have contributions to research on CA and LC-MUFAs going forward, it can be argued that *Danio rerio*, the zebrafish, may provide an attractive tradeoff between lipid metabolism, neurophysiology and ease of operation.

## Data Availability

No datasets were generated or analysed during the current study.

## Abbreviations

AD: Alzheimer’s disease
BBB: Blood-brain barrier
CA: Cetoleic acid
DHA: Docosahexaenoic acid
EA: Erucic acid
EPA: Eicosapentaenoic acids
FATP: Fatty acid transport protein
ETA: Eicosatetraenoic acid
HDL: High-density lipoprotein
LDL: Low-density lipoprotein
MUFAs: Monounsaturated fatty acids
PPAR: Peroxisome proliferator-activated receptor-signaling
PUFAs: Polyunsaturated fatty acids
SFAs: Saturated fatty acids
TAG: Triacylglycerol

## References

[1] Fahy E, Cotter D, Sud M, Subramaniam S. Lipid classification, structures and tools. Biochim Biophys Acta - Mol Cell Biol Lipids. 2011;1811:637–47. 10.1016/j.bbalip.2011.06.009 PMC3995129 21704189

[2] Furse S, Egmond MR, Killian JA. Isolation of lipids from biological samples. Mol Membr Biol. 2015;32:55–64. 26212444 10.3109/09687688.2015.1050468

[3] Custers, Emma EM, Kiliaan, Amanda J. Dietary lipids from body to brain. Prog Lipid Res. 2022;85:101144–57. 10.1016/j.plipres.2021.101144 34915080

[4] Nagle JF, Tristram-Nagle S. Structure of lipid bilayers. Biochim Biophys Acta - Rev Biomembr. 2000;1469:159–95. 10.1016/s0304-4157(00)00016-2 PMC2747654 11063882

[5] Wang S et al. Solid-state NMR spectroscopy structure determination of a lipid-embedded heptahelical membrane protein. Nat Methods2013;10:1007–12. 10.1038/nmeth.2635 24013819

[6] Hamley S. The effect of replacing saturated fat with mostly n-6 polyunsaturated fat on coronary heart disease: a meta-analysis of randomised controlled trials. Nutr J. 2017;16:1–16. 28526025 10.1186/s12937-017-0254-5 PMC5437600

[7] Nitsch RM, et al. Evidence for a membrane defect in alzheimer disease brain. Proc Natl Acad Sci U S A. 1992;89:1671–5. 1311847 10.1073/pnas.89.5.1671 PMC48514

[8] Valenza M, et al. Dysfunction of the cholesterol biosynthetic pathway in huntington’s disease. J Neurosci. 2005;25:9932–9. 16251441 10.1523/JNEUROSCI.3355-05.2005 PMC6725556

[9] Wallin A, Gottfries CG, Karlsson I, Svennerholm L. Decreased Myelin lipids in alzheimer’s disease and vascular dementia. Acta Neurol Scand. 1989;80:319–23. 2816288 10.1111/j.1600-0404.1989.tb03886.x

[10] Bazan NG, Calandria JM, Gordon WC. Docosahexaenoic acid and its derivative neuroprotectin d1 display neuroprotective properties in the retina, brain and central nervous system. Nestle Nutr Inst Workshop Ser. 2013;77:121–31. 24107502 10.1159/000351395

[11] Livingston G, et al. Dementia prevention, intervention, and care: 2020 report of the lancet commission. Lancet. 2020;396:413–46. 32738937 10.1016/S0140-6736(20)30367-6 PMC7392084

[12] Borenstein AR, Copenhaver CI, Mortimer JA. Early-life risk factors for alzheimer disease. Alzheimer Dis Assoc Disord. 2006;20:63–72. 16493239 10.1097/01.wad.0000201854.62116.d7

[13] Fischer P, et al. Risk factors for alzheimer dementia in a community-based birth cohort at the age of 75 years. Dement Geriatr Cogn Disord. 2008;25:501–7. 18446027 10.1159/000128577

[14] Luo J et al. Genetic associations between modifiable risk factors and alzheimer disease. JAMA Netw Open 6, (2023).10.1001/jamanetworkopen.2023.13734. 10.1001/jamanetworkopen.2023.13734 PMC10193187 37195665

[15] Escribá PV. Membrane-lipid therapy: A historical perspective of membrane-targeted therapies — From lipid bilayer structure to the pathophysiological regulation of cells. Biochim Biophys Acta - Biomembr. 2017;1859:1493–506. 28577973 10.1016/j.bbamem.2017.05.017

[16] Cenacchi T, et al. Cognitive decline in the elderly: A double- blind, placebo- controlled multicenter study on efficacy of phosphatidylserine administration. Aging Clin Exp Res. 1993;5:123–33. 10.1007/BF03324139 8323999

[17] Emre C, et al. Intranasal delivery of pro-resolving lipid mediators rescues memory and gamma Oscillation impairment in app NL-G-F/NL-G-F mice. Commun Biol. 2022;5:245. 35314851 10.1038/s42003-022-03169-3 PMC8938447

[18] Swanson D, Block R, Mousa SA. Omega-3 fatty acids EPA and DHA: health benefits throughout life. Adv Nutr. 2012;3:1–7. 22332096 10.3945/an.111.000893 PMC3262608

[19] WHO. W. H. O. Interim summary of conclusions and dietary recommendations on total fat & fatty acids introduction and definitions. Interim Summ 1–12 (2008).

[20] Sibbett RA, Russ TC, Deary IJ, Starr JM. Risk factors for dementia in the ninth decade of life and beyond: A study of the Lothian birth cohort 1921. BMC Psychiatry. 2017;17:205. 28578665 10.1186/s12888-017-1366-3 PMC5455126

[21] Mohaibes RJ, et al. The hydroxylated form of docosahexaenoic acid (DHA-H) modifies the brain lipid composition in a model of alzheimer’s disease, improving behavioral motor function and survival. Biochim Biophys Acta - Biomembr. 2017;1859:1596–603. 28284721 10.1016/j.bbamem.2017.02.020

[22] Guu TW, et al. International society for nutritional psychiatry research practice guidelines for Omega-3 fatty acids in the treatment of major depressive disorder. Psychother Psychosom. 2019;88:263–73. 31480057 10.1159/000502652

[23] Tsutsumi R, et al. Long-chain monounsaturated fatty acids improve endothelial function with altering microbial flora. Transl Res. 2021;237:16–30. 33775867 10.1016/j.trsl.2021.03.016

[24] Gudbrandsen OA. Effects of diets containing fish oils or fish oil concentrates with high cetoleic acid content on the Circulating cholesterol concentration in rodents. A systematic review and meta-analysis. Br J Nutr. 2024;131:606–21. 37737066 10.1017/S0007114523002118 PMC10803824

[25] Ostbye TKK, et al. The long-chain monounsaturated cetoleic acid improves the efficiency of the n-3 fatty acid metabolic pathway in Atlantic salmon and human HepG2 cells. Br J Nutr. 2019;122:755–68. 31288871 10.1017/S0007114519001478

[26] ACDLabs. ChemSketch, version 12.01. (2010).

[27] Yang ZH, Miyahara H, Iwasaki Y, Takeo J, Katayama M. Dietary supplementation with long-chain monounsaturated fatty acids attenuates obesity-related metabolic dysfunction and increases expression of PPAR gamma in adipose tissue in type 2 diabetic KK-Ay mice. Nutr Metab. 2013;10:16. 10.1186/1743-7075-10-16 PMC3570324 23360495

[28] Kattner G, Hagen W. Polar herbivorous copepods - different pathways in lipid biosynthesis. ICES J Mar Sci. 1995;52:329–35.

[29] Cooper MH, Iverson SJ, Rouvinen-Watt K. Metabolism of dietary cetoleic acid (22:1n‐11) in Mink (Mustela vison) and Gray seals (Halichoerus grypus) studied using radiolabeled fatty acids. Physiol Biochem Zool. 2006;79:820–9. 16826508 10.1086/505513

[30] Yang ZH, et al. Long-chain monounsaturated fatty acid-rich fish oil attenuates the development of atherosclerosis in mouse models. Mol Nutr Food Res. 2016;60:2208–18. 27273599 10.1002/mnfr.201600142 PMC5056854

[31] Graeve M, Albers C, Kattner G. Assimilation and biosynthesis of lipids in Arctic Calanus species based on feeding experiments with a 13 C labelled diatom. J Exp Mar Bio Ecol. 2005;317:109–25.

[32] Falk-Petersen S, Mayzaud P, Kattner G, Sargent JR. Lipids and life strategy of Arctic Calanus. Mar Biol Res. 2009;5:18–39.

[33] Albers CS, Kattner G, Hagen W. The compositions of wax esters, triacylglycerols and phospholipids in Arctic and Antarctic copepods: evidence of energetic adaptations. Mar Chem. 1996;55:347–58.

[34] Zhang JY, Kothapalli KSD, Brenna JT. Desaturase and elongase limiting endogenous long chain polyunsaturated fatty acid biosynthesis. Curr Opin Clin Nutr Metab Care. 2016;19:103. 26828581 10.1097/MCO.0000000000000254 PMC4768719

[35] Ntambi JM, Miyazaki M. Recent insights into stearoyl-CoA desaturase-1. Curr Opin Lipidol. 2003;14:255–61. 12840656 10.1097/00041433-200306000-00005

[36] FULCO AJ, MEAD JF. The biosynthesis of lignoceric, cerebronic, and nervonic acids. J Biol Chem. 1961;236:2416–20. 13702539

[37] Bazan NG, Molina MF, Gordon WC. Docosahexaenoic acid signalolipidomics in nutrition: significance in aging, neuroinflammation, macular degeneration, Alzheimer’s, and other neurodegenerative diseases. Annu Rev Nutr. 2011;31:321–51. 21756134 10.1146/annurev.nutr.012809.104635 PMC3406932

[38] Morris MC, et al. MIND diet associated with reduced incidence of alzheimer’s disease. Alzheimer’s Dement. 2015;11:1007–14. 25681666 10.1016/j.jalz.2014.11.009 PMC4532650

[39] Morris MC, et al. MIND diet slows cognitive decline with aging. Alzheimer’s Dement. 2015;11:1015–22. 26086182 10.1016/j.jalz.2015.04.011 PMC4581900

[40] Long Simon. World alzheimerreducing dementia risk: never too early, never too late. Adi 2023;1–96.

[41] Dhana K, et al. MIND Diet, common brain Pathologies, and cognition in Community-Dwelling older adults. J Alzheimer’s Dis. 2021;83:683–92. 34334393 10.3233/JAD-210107 PMC8480203

[42] Cherian L, et al. Mediterranean-Dash intervention for neurodegenerative delay (MIND) diet slows cognitive decline after stroke. J Prev Alzheimer’s Dis. 2019;6:267–73. 31686099 10.14283/jpad.2019.28 PMC7199507

[43] Hosking DE, Eramudugolla R, Cherbuin N, Anstey K. J. MIND not mediterranean diet related to 12-year incidence of cognitive impairment in an Australian longitudinal cohort study. Alzheimer’s Dement. 2019;15:581–9. 30826160 10.1016/j.jalz.2018.12.011

[44] Van Lent DM, et al. Mind diet adherence and cognitive performance in the Framingham heart study. J Alzheimer’s Dis. 2021;82:827–39. 34092629 10.3233/JAD-201238

[45] Berendsen AM, et al. Association of long-term adherence to the Mind diet with cognitive function and cognitive decline in American women. J Nutr Heal Aging. 2018;22:222–9. 10.1007/s12603-017-0909-0 PMC12876286 29380849

[46] Chen H, et al. Association of the mediterranean dietary approaches to stop hypertension intervention for neurodegenerative delay (MIND) diet with the risk of dementia. JAMA Psychiatry. 2023;80:630–8. 37133875 10.1001/jamapsychiatry.2023.0800 PMC10157510

[47] Akbaraly TN, et al. Association of midlife diet with subsequent risk for dementia. JAMA - J Am Med Assoc. 2019;321:957–68. 10.1001/jama.2019.1432 PMC6436698 30860560

[48] Liu X, et al. Mediterranean-DASH intervention for neurodegenerative delay (MIND) study: Rationale, design and baseline characteristics of a randomized control trial of the MIND diet on cognitive decline. Contemp Clin Trials. 2021;102:106270. 33434704 10.1016/j.cct.2021.106270 PMC8042655

[49] WHO GUIDELINES. Risk reduction of cognitive decline and dementia: WHO guidelines. Who (2019). 31219687

[50] Nazari E, et al. Beneficial effect of brassica Nigra fixed oil on the changes in memory caused by B-Amyloid in an animal model. Pharm Sci. 2020;26:261–9.

[51] Rahman MH, Habib K, Rahman S, Nasreen L, Ud-Daula A. Ameliorating effect of dietary Sesame oil on high erucic acid rapeseed Powder-Induced changes of blood serum lipids in rats. IOSR J Environ Sci. 2016;10:49–53.

[52] Mohanty S, Mehrotra N, Khan MT, Sharma S, Tripathi P. Paradoxical effects of erucic acid - A fatty acid with Two-Faced implications. Nutr Rev. 2025;83:2028–41. 40202517 10.1093/nutrit/nuaf032

[53] Bernasconi AA, Wiest MM, Lavie CJ, Milani RV, Laukkanen JA. Effect of Omega-3 dosage on cardiovascular outcomes: an updated Meta-Analysis and Meta-Regression of interventional trials. Mayo Clin Proc. 2021;96:304–13. 32951855 10.1016/j.mayocp.2020.08.034

[54] Djuricic I, Calder PC. Beneficial outcomes of omega-6 and omega-3 polyunsaturated fatty acids on human health: an update for 2021. Nutrients. 2021;13:2421. 34371930 10.3390/nu13072421 PMC8308533

[55] Best KP, Gibson RA, Makrides M. ISSFAL statement number 7 – Omega-3 fatty acids during pregnancy to reduce preterm birth. Prostaglandins Leukot Essent Fat Acids. 2022;186:102495. 10.1016/j.plefa.2022.102495 36228573

[56] Innes JK, Calder PC. The differential effects of eicosapentaenoic acid and docosahexaenoic acid on cardiometabolic risk factors: A systematic review. Int J Mol Sci. 2018;19:532. 29425187 10.3390/ijms19020532 PMC5855754

[57] Tobin D, Midtbø LK, Mildenberger J, Svensen H, Stoknes I. The effect of fish oil rich in cetoleic acid on the omega-3 index and skin quality. Prostaglandins Leukot Essent Fat Acids. 2024;201:102616. 10.1016/j.plefa.2024.102616 38788345

[58] Nookaew I, Gabrielsson BG, Holmäng A, Sandberg AS, Nielsen J. Identifying molecular effects of diet through systems biology: influence of herring diet on sterol metabolism and protein turnover in mice. PLoS ONE. 2010;5:e12361. 20808764 10.1371/journal.pone.0012361 PMC2927425

[59] Yang ZH, Miyahara H, Takemura S, Hatanaka A. Dietary Saury oil reduces hyperglycemia and hyperlipidemia in diabetic KKAy mice and in diet-induced obese C57BL/6J mice by altering gene expression. Lipids. 2011;46:425–34. 21465306 10.1007/s11745-011-3553-1

[60] Yang ZH, et al. Long-term dietary supplementation with Saury oil attenuates metabolic abnormalities in mice fed a high-fat diet: combined beneficial effect of omega-3 fatty acids and long-chain monounsaturated fatty acids. Lipids Health Dis. 2015;14:155. 26627187 10.1186/s12944-015-0161-8 PMC4666194

[61] Yang ZH, Emma-Okon B, Remaley AT. Dietary marine-derived long-chain monounsaturated fatty acids and cardiovascular disease risk: a mini review. Lipids Health Dis. 2016;15:1–9. 27876051 10.1186/s12944-016-0366-5 PMC5120510

[62] Matsumoto C, Matthan NR, Lichtenstein AH, Gaziano M, J., Djoussé L. Red blood cell MUFAs and risk of coronary artery disease in the physicians’ health study. Am J Clin Nutr. 2013;98:749–54. 23824727 10.3945/ajcn.113.059964 PMC3743735

[63] Abete I, Goyenechea E, Zulet MA, Martínez JA. Obesity and metabolic syndrome: potential benefit from specific nutritional components. Nutr Metab Cardiovasc Dis. 2011;21:B1–15. 21764273 10.1016/j.numecd.2011.05.001

[64] Gillingham LG, Harris-Janz S, Jones PJH. Dietary monounsaturated fatty acids are protective against metabolic syndrome and cardiovascular disease risk factors. Lipids. 2011;46:209–28. 21308420 10.1007/s11745-010-3524-y

[65] Yang ZH, et al. Supplementation with Saury oil, a fish oil high in omega-11 monounsaturated fatty acids, improves plasma lipids in healthy subjects. J Clin Lipidol. 2020;14:53–e652. 31784345 10.1016/j.jacl.2019.10.013 PMC8336206

[66] Gabrielsson BG, et al. Dietary herring improves plasma lipid profiles and reduces atherosclerosis in obese low-density lipoprotein receptor-deficient mice. Int J Mol Med. 2012;29:331–7. 22160183 10.3892/ijmm.2011.856

[67] Golovko MY, Murphy EJ. Uptake and metabolism of plasma-derived erucic acid by rat brain. J Lipid Res. 2006;47:1289–97. 16525189 10.1194/jlr.M600029-JLR200

[68] Kramer JKG, Sauer FD, Wolynetz MS, Farnworth ER, Johnston KM. Effects of dietary saturated fat on erucic acid induced myocardial lipidosis in rats. Lipids. 1992;27:619–23. 1383668 10.1007/BF02536120

[69] Emken EA, Adlof RO, Duval SM, Nelson GJ. Effect of dietary docosahexaenoic acid on desaturation and uptake *in vivo* of isotope-labeled oleic, linoleic, and linolenic acids by male subjects. Lipids. 1999;34:785–91. 10529088 10.1007/s11745-999-0424-2

[70] Domenichiello AF, et al. Whole-body docosahexaenoic acid synthesis-secretion rates in rats are constant across a large range of dietary α-linolenic acid intakes. J Nutr. 2017;147:37–44. 27852871 10.3945/jn.116.232074

[71] Keller H, et al. Fatty acids and retinoids control lipid metabolism through activation of peroxisome proliferator-activated receptor-retinoid X receptor heterodimers. Proc Natl Acad Sci U S A. 1993;90:2160–4. 8384714 10.1073/pnas.90.6.2160 PMC46045

[72] Langelier B, Furet JP, Perruchot MH, Alessandri JM. Docosahexaenoic acid membrane content and mRNA expression of acyl-CoA oxidase and of peroxisome proliferator-activated receptor-δ are modulated in Y79 retinoblastoma cells differently by low and high doses of α-linolenic acid. J Neurosci Res. 2003;74:134–41. 13130515 10.1002/jnr.10714

[73] Edvardsson U, Ljungberg A, Oscarsson J. Insulin and oleic acid increase PPARγ2 expression in cultured mouse hepatocytes. Biochem Biophys Res Commun. 2006;340:111–7. 16364246 10.1016/j.bbrc.2005.12.008

[74] Hsu SC, Huang CJ. Reduced fat mass in rats fed a high oleic acid-rich safflower oil diet is associated with changes in expression of hepatic PPARα and adipose SREBP-1c-regulated genes. J Nutr. 2006;136:1779–85. 16772437 10.1093/jn/136.7.1779

[75] Altinoz MA, Ozpinar A. PPAR-δ and erucic acid in multiple sclerosis and alzheimer’s Disease. Likely benefits in terms of immunity and metabolism. Int Immunopharmacol. 2019;69:245–56. 30738994 10.1016/j.intimp.2019.01.057

[76] Gervois P, Torra IP, Fruchart JC, Staels B. Regulation of lipid and lipoprotein metabolism by PPAR activators. Clin Chem Lab Med. 2000;38:3–11. 10774955 10.1515/CCLM.2000.002

[77] Halvorsen B, et al. Effects of long-chain monounsaturated and n-3 fatty acids on fatty acid oxidation and lipid composition in rats. Ann Nutr Metab. 2001;45:30–7. 11244185 10.1159/000046703

[78] Sprecher H. Metabolism of highly unsaturated n-3 and n-6 fatty acids. Biochim Biophys Acta - Mol Cell Biol Lipids. 2000;1486:219–31. 10.1016/s1388-1981(00)00077-9 10903473

[79] Opstvedt J. Fish lipids: more than n-3 fatty acids? Med Hypotheses. 1997;48:481–3. 9247889 10.1016/s0306-9877(97)90115-8

[80] Østbye T-KK, et al. The long-chain monounsaturated cetoleic acid improves the efficiency of the n-3 fatty acid metabolic pathway in Atlantic salmon and human HepG2 cells. Br J Nutr. 2019;122:755–68. 31288871 10.1017/S0007114519001478

[81] Serhan CN, Chiang N. Resolvins and cysteinyl-containing pro-resolving mediators activate resolution of infectious inflammation and tissue regeneration. Prostaglandins Other Lipid Mediat. 2023;166:106718. 36813255 10.1016/j.prostaglandins.2023.106718 PMC10175197

[82] Farag MA, Gad MZ. Omega-9 fatty acids: potential roles in inflammation and cancer management. J Genet Eng Biotechnol. 2022;20:48. 35294666 10.1186/s43141-022-00329-0 PMC8927560

[83] Reid MM, et al. NPD1 plus RvD1 mediated ischemic stroke penumbra protection increases expression of Pro-homeostatic microglial and astrocyte genes. Cell Mol Neurobiol. 2023;43:3555–73. 37270727 10.1007/s10571-023-01363-3 PMC10477115

[84] Sun GY, Xu J, Jensen MD, Simonyi A. Phospholipase A2 in the central nervous system: implications for neurodegenerative diseases. J Lipid Res. 2004;45:205–13. 14657205 10.1194/jlr.R300016-JLR200

[85] Dobri AM, Dudău M, Enciu AM, Hinescu ME. CD36 in alzheimer’s disease: an overview of molecular mechanisms and therapeutic targeting. Neuroscience. 2021;453:301–11. 33212223 10.1016/j.neuroscience.2020.11.003

[86] Zhang W, et al. Fatty acid transporting proteins: roles in brain development, aging, and stroke. Prostaglandins Leukot Essent Fat Acids. 2018;136:35–45. 10.1016/j.plefa.2017.04.004 PMC5650946 28457600

[87] Shioda N, et al. Heart-type fatty acid binding protein regulates dopamine D2 receptor function in mouse brain. J Neurosci. 2010;30:3146–55. 20181611 10.1523/JNEUROSCI.4140-09.2010 PMC6633935

[88] Nguyen LN, et al. Mfsd2a is a transporter for the essential omega-3 fatty acid docosahexaenoic acid. Nature. 2014;509:503–6. 24828044 10.1038/nature13241

[89] Pélerin H, et al. Gene expression of fatty acid transport and binding proteins in the blood-brain barrier and the cerebral cortex of the rat: differences across development and with different DHA brain status. Prostaglandins Leukot Essent Fat Acids. 2014;91:213–20. 10.1016/j.plefa.2014.07.004 25123062

[90] Tracey TJ, Steyn FJ, Wolvetang EJ, Ngo ST. Neuronal lipid metabolism: multiple pathways driving functional outcomes in health and disease. Front Mol Neurosci. 11, (2018). 10.3389/fnmol.2018.00010 PMC5787076 29410613

[91] Mitchell RW, On NH, Bigio D, Miller MR, D. W., Hatch GM. Fatty acid transport protein expression in human brain and potential role in fatty acid transport across human brain microvessel endothelial cells. J Neurochem. 2011;117:735–46. 21395585 10.1111/j.1471-4159.2011.07245.x

[92] Coet NR, Smith AJ, Frohnert BI, Watkins PA, Bernlohr DA. The fatty acid transport protein (FATP1) is a very long chain acyl-CoA synthetase. J Biol Chem. 1999;274:36300–4. 10593920 10.1074/jbc.274.51.36300

[93] Ioannou MS. Current insights into fatty acid transport in the brain. J Membr Biol. 2020;253:375–9. 32968835 10.1007/s00232-020-00140-3

[94] Guemez-Gamboa A, et al. Inactivating mutations in MFSD2A, required for omega-3 fatty acid transport in brain, cause a lethal microcephaly syndrome. Nat Genet. 2015;47:809–13. 26005868 10.1038/ng.3311 PMC4547531

[95] Arellanes IC, et al. Brain delivery of supplemental docosahexaenoic acid (DHA): A randomized placebo-controlled clinical trial. EBioMedicine. 2020;59:102883. 32690472 10.1016/j.ebiom.2020.102883 PMC7502665

[96] Jakubec M, et al. Plasma-derived exosome-like vesicles are enriched in lyso-phospholipids and pass the blood-brain barrier. PLoS ONE. 2020;15:e0232442. 32956358 10.1371/journal.pone.0232442 PMC7505448

[97] Belayev L, Reid M, Bazan N. Novel lipid mediators as a promising therapeutic strategy for ischemic stroke. Med Res Arch 2023;11:1–8. 10.18103/mra.v11i1.3333 PMC9910361 36777192

[98] Bermúdez MA, et al. Roles of palmitoleic acid and its positional Isomers, hypogeic and Sapienic acids, in Inflammation, metabolic diseases and cancer. Cells. 2022;11:2146. 35883589 10.3390/cells11142146 PMC9319324

[99] Lauritzen L, Hansen HS, Jorgensen MH, Michaelsen KF. The essentiality of long chain n-3 fatty acids in relation to development and function of the brain and retina. Prog Lipid Res. 2001;40:1–94. 11137568 10.1016/s0163-7827(00)00017-5

[100] Galán-Arriero I, et al. The role of Omega-3 and Omega-9 fatty acids for the treatment of neuropathic pain after neurotrauma. Biochim Biophys Acta - Biomembr. 2017;1859:1629–35. 28495596 10.1016/j.bbamem.2017.05.003

[101] Gordon WC, Bazan NG. Mediator lipidomics in ophthalmology: targets for modulation in inflammation, neuroprotection and nerve regeneration. Curr Eye Res. 2013;38:995–1005. 23981028 10.3109/02713683.2013.827211

[102] Anderson RE, et al. Low docosahexaenoic acid levels in rod outer segments of rats with P23H and S334ter rhodopsin mutations. Mol Vis. 2002;8:351–8. 12355064

[103] Orban T, et al. Serum levels of lipid metabolites in age-related macular degeneration. FASEB J. 2015;29:4579–88. 26187344 10.1096/fj.15-275289 PMC4608905

[104] Hoffman DR, Birch DG. KARGER,. Omega 3 fatty acid status in patients with retinitis pigmentosa. in *World review of nutrition and dietetics* vol. 83 52–60 (1998). 10.1159/000059653 9648504

[105] Liu A, Chang J, Lin Y, Shen Z, Bernstein PS. Long-chain and very long-chain polyunsaturated fatty acids in ocular aging and age-related macular degeneration. J Lipid Res. 2010;51:3217–29. 20688753 10.1194/jlr.M007518 PMC2952562

[106] Yeboah GK, Lobanova ES, Brush RS, Agbaga MP. Very long chain fatty acid-containing lipids: A decade of novel insights from the study of ELOVL4. J Lipid Res. 2021;62:100030. 33556440 10.1016/j.jlr.2021.100030 PMC8042400

[107] Shariati SAM, De Strooper B. Redundancy and divergence in the amyloid precursor protein family. FEBS Lett. 2013;587:2036–45. 23707420 10.1016/j.febslet.2013.05.026

[108] Amtul Z, Uhrig M, Supino R, Beyreuther K. Phospholipids and a phospholipid-rich diet alter the *in vitro* amyloid-beta peptide levels and amyloid-beta 42/40 ratios. Neurosci Lett. 2010;481:73–7. 20600609 10.1016/j.neulet.2010.06.046

[109] Grimm MOW, Rothhaar TL, Hartmann T. The role of APP proteolytic processing in lipid metabolism. Exp Brain Res. 2012;217:365–75. 22179528 10.1007/s00221-011-2975-6

[110] Grimm MOW, Mett J, Grimm HS, Hartmann TAPP. Function and lipids: A bidirectional link. Front Mol Neurosci 2017;10:1–18. 10.3389/fnmol.2017.00063 PMC5344993 28344547

[111] Linden DJ, Murakami K, Routtenberg A. A newly discovered protein kinase C activator (oleic acid) enhances long-term potentiation in the intact hippocampus. Brain Res. 1986;379:358–63. 3091192 10.1016/0006-8993(86)90790-0

[112] Dawkins E et al. Membrane lipid remodeling modulates γ-secretase processivity. J Biol Chem 2023;299:103027–39. 10.1016/j.jbc.2023.103027 PMC10070668 36805335

[113] Cullen N, et al. Association of CSF Aβ38Levels with risk of alzheimer Disease-Related decline. Neurology. 2022;98:E958–67. 34937781 10.1212/WNL.0000000000013228 PMC8901176

[114] Hashimoto M, et al. Docosahexaenoic acid provides protection from impairment of learning ability in alzheimer’s disease model rats. J Neurochem. 2002;81:1084–91. 12065621 10.1046/j.1471-4159.2002.00905.x

[115] Geng X, et al. Effects of docosahexaenoic acid and its peroxidation product on Amyloid-β Peptide-Stimulated microglia. Mol Neurobiol. 2020;57:1085–98. 31677009 10.1007/s12035-019-01805-4 PMC8080969

[116] Wang X et al. Docosahexaenoic Acid-Acylated Astaxanthin monoester ameliorates Amyloid-β pathology and neuronal damage by restoring autophagy in alzheimer’s disease models. Mol Nutr Food Res 2024;68:2300414–27. 10.1002/mnfr.202300414 37991232

[117] Green KN, et al. Dietary docosahexaenoic acid and docosapentaenoic acid ameliorate amyloid-β and Tau pathology via a mechanism involving presenilin 1 levels. J Neurosci. 2007;27:4385–95. 17442823 10.1523/JNEUROSCI.0055-07.2007 PMC6672302

[118] Grimm MOW, et al. Docosahexaenoic acid reduces amyloid β production via multiple pleiotropic mechanisms. J Biol Chem. 2011;286:14028–39. 21324907 10.1074/jbc.M110.182329 PMC3077603

[119] Grimm MOW, et al. Eicosapentaenoic acid and docosahexaenoic acid increase the degradation of amyloid-β by affecting insulin-degrading enzyme1. Biochem Cell Biol. 2016;94:534–42. 27813426 10.1139/bcb-2015-0149

[120] Mi-Mba MFOM, et al. Differential impact of eicosapentaenoic acid and docosahexaenoic acid in an animal model of alzheimer’s disease. J Lipid Res. 2024;65:100682. 39490923 10.1016/j.jlr.2024.100682 PMC11650307

[121] Shevlyakov AD, et al. Zebrafish as a promising experimental model of traumatic brain injury. J Evol Biochem Physiol. 2024;60:594–611.

[122] Pang K, et al. An app knock-in rat model for alzheimer’s disease exhibiting Aβ and Tau pathologies, neuronal death and cognitive impairments. Cell Res. 2022;32:157–75. 34789895 10.1038/s41422-021-00582-x PMC8807612

[123] Sari G, et al. A mouse model of humanized liver shows a human-like lipid profile, but does not form atherosclerotic plaque after Western type diet. Biochem Biophys Res Commun. 2020;524:510–5. 32014257 10.1016/j.bbrc.2020.01.067

[124] Alexander AG, Marfil V, Li C. Use of C. elegans as a model to study alzheimer’s disease and other neurodegenerative diseases. Front Genet. 2014;5:279. 25250042 10.3389/fgene.2014.00279 PMC4155875

[125] Dunton AD, Göpel T, Ho DH, Burggren W. Form and function of the vertebrate and invertebrate blood-brain barriers. Int J Mol Sci 22, (2021). 10.3390/ijms222212111 PMC8618301 34829989

[126] Jeibmann A, Paulus W. Drosophila melanogaster as a model organism of brain diseases. Int J Mol Sci. 2009;10:407–40. 19333415 10.3390/ijms10020407 PMC2660653

[127] Beauchamp A et al. Whole-brain comparison of rodent and human brains using spatial transcriptomics. *Elife* 11, (2022). 10.7554/eLife.79418 PMC9708081 36342372

[128] Tharp WG, Sarkar. I. N. Origins of amyloid-β. BMC Genomics. 2013;14:290. 23627794 10.1186/1471-2164-14-290 PMC3660159

[129] Khan AA, Ali RH, Mirza B. Evolutionary history of alzheimer Disease-Causing protein family presenilins with pathological implications. J Mol Evol. 2020;88:674–88. 33001284 10.1007/s00239-020-09966-w

[130] Southan C, Hancock JM. A Tale of two drug targets: the evolutionary history of BACE1 and BACE2. Front Genet. 2013;4:293. 24381583 10.3389/fgene.2013.00293 PMC3865767

[131] Seynnaeve D et al. Recent insights on alzheimer’s disease originating from yeast models. Int J Mol Sci 2018;19:1947–72. 10.3390/ijms19071947 PMC6073265 29970827

[132] Brown L, Panchal SK. Rodent models for metabolic syndrome research. *J. Biomed. Biotechnol.* 2011, 351982 (2011). 10.1155/2011/351982 PMC3018657 21253582

[133] Zoccolan D, Oertelt N, DiCarlo JJ, Cox DD. A rodent model for the study of invariant visual object recognition. *Proc. Natl. Acad. Sci. U. S. A.* 2009;106:8748–53. 10.1073/pnas.0811583106 PMC2679579 19429704

[134] Mathiasen JR, DiCamillo A. Social recognition assay in the rat. Curr Protoc Neurosci Chapter *8*, Unit 8.5I (2010). 10.1002/0471142301.ns0805is53 20938925

[135] Fonseka TM, Wen XY, Foster JA, Kennedy SH. Zebrafish models of major depressive disorders. J Neurosci Res. 2016;94:3–14. 26452974 10.1002/jnr.23639

[136] Gilbert MJH, Zerulla TC, Tierney KB. Zebrafish (Danio rerio) as a model for the study of aging and exercise: physical ability and trainability decrease with age. Exp Gerontol. 2013;50:106–13. 24316042 10.1016/j.exger.2013.11.013

[137] Wullimann MF, Mueller T. Teleostean and mammalian forebrains contrasted: evidence from genes to behavior. J Comp Neurol. 2004;475:143–62. 15211457 10.1002/cne.20183

[138] Vance JE. Dysregulation of cholesterol balance in the brain: contribution to neurodegenerative diseases. DMM Dis Model Mech. 2012;5:746–55. 23065638 10.1242/dmm.010124 PMC3484857

[139] Howe K, et al. The zebrafish reference genome sequence and its relationship to the human genome. Nature. 2013;496:498–503. 23594743 10.1038/nature12111 PMC3703927

[140] Wu K, et al. Phylogeny and expression of ADAM10 and ADAM17 homologs in lamprey. Fish Physiol Biochem. 2023;49:321–34. 36964830 10.1007/s10695-023-01184-7

[141] Blais EM, et al. Reconciled rat and human metabolic networks for comparative toxicogenomics and biomarker predictions. Nat Commun. 2017;8:14250. 28176778 10.1038/ncomms14250 PMC5309818

[142] Feldmann HM, Golozoubova V, Cannon B, Nedergaard J. UCP1 ablation induces obesity and abolishes Diet-Induced thermogenesis in mice exempt from thermal stress by living at thermoneutrality. Cell Metab. 2009;9:203–9. 19187776 10.1016/j.cmet.2008.12.014

[143] Zang L, Maddison LA, Chen W. Zebrafish as a model for obesity and diabetes. Front Cell Dev Biol 2018;6:1–13. 10.3389/fcell.2018.00091 PMC6110173 30177968

[144] Smolińska K, et al. Innovative high fat diet establishes a novel zebrafish model for the study of visceral obesity. Sci Rep. 2024;14:1–14. 38321127 10.1038/s41598-024-53695-9 PMC10847117

[145] Bowley G, et al. Zebrafish as a tractable model of human cardiovascular disease. Br J Pharmacol. 2022;179:900–17. 33788282 10.1111/bph.15473

[146] Hölttä-Vuori M, et al. Zebrafish: gaining popularity in lipid research. Biochem J. 2010;429:235–42. 20578994 10.1042/BJ20100293

[147] Cutest Systems Ltd Cardiff. U. K. A study to evaluate the effect of fish oil concentrate on skin function. https://clinicaltrials.gov/study/NCT07133477.

[148] Helse Møre og Romsdal HF. Ålesund, Møre Og Romsdal, Norway, 6017. Nutritional study to determine the effect of fish oil on atopic dermatitis. https://clinicaltrials.ov/study/NCT06194045.

[149] Hospital OU, Oslo N. Effects of Cetoleic Acid on Atherosclerosis (Ketolinsyre’s Effekt på Aterosklerose). https://clinicaltrials.gov/study/NCT06172335.

[150] University of Oslo. Oslo, Norway, 0316. Health effects of cetoleic acid (A randomized double blinded controlled Trial). https://clinicaltrials.gov/study/NCT04841044.

[151] University of Bergen. Bergen, N. Effect of herring oil concentrate on LDL cholesterol concentration in adults. https://clinicaltrials.gov/study/NCT06364163.

[152] Institutt for Medisinsk Biologi. UiT Norges arktiske universitet, Tromsø, N. & KG Jebsen-senter for Hjertetrening, NTNU, Trondheim, N. Calanus Oil Supplementation and Maximal Oxygen Uptake. https://clinicaltrials.gov/study/NCT02908828.

[153] University of Tromso. Tromsø, Norway, 9037. Effect of Calanus Oli on Intra-abdominal Fat, Glucose Tolerance and Lipids in Man. https://clinicaltrials.gov/study/NCT01193543.

